# Meteorite Impact-Induced Rapid NH_3_ Production on Early Earth: *Ab Initio* Molecular Dynamics Simulation

**DOI:** 10.1038/srep38953

**Published:** 2016-12-14

**Authors:** Kohei Shimamura, Fuyuki Shimojo, Aiichiro Nakano, Shigenori Tanaka

**Affiliations:** 1 Graduate School of System Informatics, Kobe University, 1-1 Rokkodai, Nada-ku, Kobe 657-8501, Japan; 2 Department of Physics, Kumamoto University, 2-39-1 Kurokami, Chuo-ku, Kumamoto 860-8555, Japan; 3 Collaboratory for Advanced Computing and Simulations, Department of Physics & Astronomy, Department of Computer Science, Department of Chemical Engineering & Materials Science, and Department of Biological Sciences, University of Southern California, Los Angeles, CA 90089-0242, USA

## Abstract

NH_3_ is an essential molecule as a nitrogen source for prebiotic amino acid syntheses such as the Strecker reaction. Previous shock experiments demonstrated that meteorite impacts on ancient oceans would have provided a considerable amount of NH_3_ from atmospheric N_2_ and oceanic H_2_O through reduction by meteoritic iron. However, specific production mechanisms remain unclear, and impact velocities employed in the experiments were substantially lower than typical impact velocities of meteorites on the early Earth. Here, to investigate the issues from the atomistic viewpoint, we performed multi-scale shock technique-based *ab initio* molecular dynamics simulations. The results revealed a rapid production of NH_3_ within several picoseconds after the shock, indicating that shocks with greater impact velocities would provide further increase in the yield of NH_3_. Meanwhile, the picosecond-order production makes one expect that the important nitrogen source precursors of amino acids were obtained immediately after the impact. It was also observed that the reduction of N_2_ proceeded according to an associative mechanism, rather than a dissociative mechanism as in the Haber-Bosch process.

An enormous amount of NH_3_ is synthesized daily from chemically inert N_2_ by Haber-Bosch process and by enzymatic catalysis of nitrogenase in nitrogen fixing bacterial^1^. This is because NH_3_ is a fundamental nitrogen source for life on the current Earth. NH_3_ would also have been an essential precursor for the terrestrial amino acid synthesis such as in the Strecker reaction^2^ on the prebiotic Earth. Since N_2_ was also a main nitrogen source on the early Earth^3^,^4^, the question of how large amounts of NH_3_ were produced without the nitrogen fixation mechanisms above is an important issue concerning the origin of life. Several hypotheses have been proposed to describe reduction processes of terrestrial N_2_: reduction of NO^2−^ and NO^3−^ by oceanic Fe^2+^ (where the nitrogen oxides are assumed to have been formed from atmospheric N_2_ by electronic discharge)^5^; photoreduction of atmospheric N_2_ on the mineral surfaces^6^; reduction of crustal N_2_ on the mineral surfaces around submarine hydrothermal systems^7^,^8^. In addition, direct extraterrestrial delivery of NH_3_ might have also been made. During the periods of Late Heavy Bombardment (LHB)^9^,^10^,^11^, although dominant types of impactors (*e.g.* comets or asteroids) are still unclear, numerous impactors that contain a large amount of organic matter continued to hit the Earth^12^. From the standpoint that many comets reached the Earth, the possibility of their soft landing on the early Earth has been discussed^13^ because it has been found that the cometary dusts contain NH_3_ and other important biomolecule sources^14^,^15^,^16^.

Alternatively, such a meteorite impact on the planetary surface would have generated a shock wave and caused a sudden increase in pressure and temperature. This in turn would have induced chemical interactions among meteoritic materials such as irons, atmosphere, and ocean. In fact, previous experimental and theoretical works have reported the production of various reductive volatiles from inorganic molecules, by simulating the impact events on the early Earth^17^,^18^,^19^,^20^,^21^,^22^,^23^,^24^,^25^. Under such a circumstance, Nakazawa *et al*.^26^ have experimentally demonstrated the production of a large amount of NH_3_ under shock in a simple starting material consisting of metallic iron, N_2_, and H_2_O, even with a much smaller collision energy than expected in the actual impact. Their experiments were carried out from the standpoint that metallic iron-rich asteroids dominated during the LHB periods based on previous studies. For example, Bottke *et al*.^11^ suggested that impactors during the LHB periods originated from the E-belt existing in the periphery of the Mars-crossing zone. Majority of the E-belt asteroids would have acquired orbits similar to those of the Hungaria asteroids, which contain a large amount of metallic iron. According to a rough estimate using the observed nitrogen conversion rate from the experiment by Nakazawa *et al*.^26^, the product amount during the LHB periods reaches 1.08 × 10^7^ tons yr^−1^ (see Supplementary Information), which corresponds to ~7 % of the current annual production amount by the Haber-Bosch process (1.59 × 10^8^ tons yr^−1^)^27^. Therefore, in addition to other production mechanisms and extraterrestrial delivery previously described, the meteorite impacts could have provided an adequate amount of NH_3_ to maintain biological activities.

While this shock-induced NH_3_ production mechanism is plausible, a number of fundamental issues remain unsolved. First, it is unclear when NH_3_ was produced, *i.e.*, just after the shock or in the subsequent cooling process. Second, what is the specific reducing mechanism of N_2_? In addition, impact velocities of the experiments were much lower^26^ (~1 km/s) than typical impact velocities of meteorites^28^ (above 10 km/s), and the possibility of further increase in the production amount for higher-energy impacts remains to be examined. Since Nakazawa *et al*.^25^ have recently succeeded in producing nine types of proteinogenic amino acids and of two types of nucleobases under shock in a sample including NH_3_ as nitrogen sources, elucidation of the shock-induced NH_3_ production processes is quite important in that leads to an understanding of production mechanisms for important nitrogen precursors of fundamental biomolecules such as amino acids.

In order to study these issues from the atomistic viewpoint, we performed *ab initio* molecular dynamics (AIMD) simulations in the framework of density functional theory (DFT)^29^ in conjunction with multi-scale shock technique (MSST-AIMD)^30^. AIMD follows the trajectories of all atoms while computing interatomic interactions quantum mechanically based on the Hellmann-Feynman theorem^31^ and can therefore describe chemical reactions accurately. MSST is a simulation method based on MD and Navier-Stokes equations to model the propagation of steady shock waves for compressible flow. MSST allows simulations with fewer atoms and lower computational cost because the MD super cell follows a small Lagrangian point rather than describing the entire shock structure. Goldman *et al*.^32^,^33^ have successfully demonstrated using MSST-AIMD and density functional tight binding based MSST simulations that proteinogenic amino acid glycine and precursors for amino acid, sugar, and nucleotide syntheses such as hydrogen cyanides (HCN), formic acids (HCOOH), and formaldehydes (H_2_CO) could be formed from shocked cometary components such as NH_3_, H_2_O, CO_2_, CO, and CH_3_OH. This work suggests that the MSST method is effective in studying shock-wave-induced chemical synthesis of organic molecules. In this work, we focused on the chemical reactions that occurred in the early stage within several picoseconds after shock. Our MSST-AIMD simulations show rapid NH_3_ production under somewhat higher pressure and temperature conditions than those in the experiment as described below, where we also estimate NH_3_ production amount from the standpoint that metallic iron-rich asteroids dominated during the LHB periods. In addition, simulation results also show that the production of NH_3_ proceeds according to an associative mechanism^34^,^35^ as seen in the catalyst of nitrogenase enzyme. By analogy with the Haber-Bosch process in the usage of iron catalysts and high pressure and temperature conditions, Nakazawa *et al*.^26^ conjectured that a dissociative mechanism^34^,^35^ would be responsible for NH_3_ production in their experiments, where, in contrast to the associative mechanism, hydrogenation of N atoms occurs after a N-N triple bond has been dissociated in N_2_.

Figure 1(a) shows the initial atomic configuration. The system consisted of a Fe_36_ slab, 16 N_2_, and 38 H_2_O molecules (a total of 182 atoms) in a rectangular supercell of dimensions 29.72 Å × 8.580 Å × 8.580 Å under periodic boundary conditions. This system entails initial reactions when a meteorite collides against the ocean surface with engulfing atmospheric N_2_. The atomic configuration was prepared as follows: A Fe slab in 2 × 3 × 3 bcc unit cells was arranged in the center of supercell, where the slab has only two surfaces perpendicular to the *x* direction, and then was immersed in liquid water. After 16 H_2_O molecules are randomly replaced by N_2_ molecules (so that the ratio of Fe atoms, N atoms, and H_2_O molecules nearly coincided with that in the experimental starting material^26^), structural optimization was performed to make axial stresses vanish. Although the surfaces of the Fe slab became heterogeneous, it is reasonable because the original meteorites’ surfaces would have some disorders due to ablation at high temperature. Note that several N_2_ and H_2_O molecules were adsorbed on the slab surfaces as shown in Fig. 1(b–d and e), respectively. Using this atomic configuration, two MSST-AIMD simulations were performed, in which shock waves propagated in the *x* direction with shock speeds of 5 and 4 km/s. As will be described later, the shock speeds were set to reproduce the experimental conditions. Simulations were performed for the time duration of 4 ps. Computational details for our MSST-AIMD simulations are described in Methods.

## Results

### Shock Speed Dependence of NH_3_ Production Amount

Figure 2(a–d) show the volume ratio (*V/V*_0_ with *V*_0_ being the initial volume), pressure (*P*), particle velocity (*U*_p_), and temperature (*T*) as a function of time for 5 km/s shock-wave simulation. For all the four quantities, there are rapid changes at around 0.3 ps. The respective values of *V/V*_0_, *P*, and *U*_p_ converge to 0.58, 27.6 GPa, and 2.30 km/s within 4 ps. In contrast, the temperature rapidly increases at around 0.3 ps, and subsequently it gradually increases from around 1,500 to 2,100 K. This indicates that some exothermic reactions occur. Figure 2(e and f) show time evolution of the number of H-O, O-Fe, H-N, and H-Fe bonds. To calculate the numbers of those bonds, a bond was defined between two atoms that were within the cutoff length continuously for a prescribed lifetime. The lifetime was chosen to be 2.42 fs, and the cutoff lengths H-O, O-Fe, H-N, and H-Fe bonds were 1.25, 1.50, 2.00, and 2.50 Å, respectively. The cutoff lengths were determined from the first minima of partial radial distribution functions obtained from 5 km/s shock-wave simulation. At around 0.3 ps, a large number of dehydrogenation of H_2_O molecules (decrease of H-O bonds) are accompanied by the oxidation of the Fe slab as the number of O-Fe bond increases. While some of released H atoms exist as single atoms on the surfaces or in the interior of the Fe slab (H-Fe), the rest form covalent bonds with N atoms (H-N). Such hydrogenation of N atoms resulted in the production of three NH_3_ molecules at 1.343, 2.044, and 3.674 ps, as shown in Fig. 2(g). (This shows *cumulative quantity* because the produced NH_3_ became ammonium ion (NH_4_^+^) immediately as described below). It is thus found that NH_3_ would be produced within picoseconds with high pressure and temperature. For comparison, the experimental values of the pressure, impact velocity (which is comparable to particle velocity), temperature are ~20 GPa, ~1 km/s and ~1,700 K, respectively^26^, which are somewhat less than those of the present simulation. Although the experimental condition is closer to physical values obtained in 4 km/s shock-wave simulation, where *P* = 15.0 GPa, *U*_p_ = 1.54 km/s, and *T* = 1,200 K (see Supplementary Fig. S1(a–d)), no NH_3_ was observed within this simulation time. Taking also into account the difference in the number of H-O, O-Fe, H-N, and H-Fe bonds at 4 ps (which are 43, 47, 24, and 18 for 5 km/s shock-wave simulation, and 63, 27, 10, and 12 for 4 km/s shock-wave simulation; see Fig. 2(f) and Supplementary Fig. S1(f)), this is due to that higher pressure and temperature in 5 km/s shock-wave simulation increased the reactivity and accelerated the production reaction as described in the following. This result indicates that applying shocks with greater impact velocities would lead to the rapid NH_3_ production and increase in the yield of its product. Since a quenching process after shock compression^32^,^33^ should be reproduced for an estimation of accurate production amount of NH_3_, we will perform additional AIMD simulations with a timescale of 100 ps as a future work. However, the rapid production of NH_3_ during shock compression would be also quite important. In previous shock studies^25^,^32^ that have been successful in producing amino acids, NH_3_ was assumed to exist before the meteorite impact (*e.g.* those dissolved in the sea or included in comets). The picosecond-order production observed in our simulation makes one expect amino acids produced at meteoritic impact events without pre-existing NH_3_. In addition, the annual production amount of NH_3_ during shock compression is estimated to be about 4.3 × 10^7^ tons (see Supplementary Information), which is larger than the estimated annual production amount from the shock experiment by Nakazawa *et al*. (1.08 × 10^7^ tons yr^−1^). This also implies that shocks with greater impact velocities would increase the yield of NH_3_.

It is worth mentioning that there is also a view that the range of impact velocities in our simulations (*i.e.*, 1–3 km/s) is most realistic. Although it is much lower than the typical meteoritic impact velocity (above 10 km/s), the typical velocity does not take into account the effects of aerobraking by Earth’s atmosphere^36^ and of deceleration of breakup while passing through the atmosphere^37^. Even if the initial velocity before the atmospheric entry was higher than 10 km/s, considering these effects, the impact velocity on the planetary surface could become around 1 km/s^37^, which is comparable to our simulation condition. However, it should be also noted that the effects may strongly depend on the atmospheric density. Since the density of prebiotic atmosphere is also still unknown, the deceleration effects would become weaker if the density was lower than that assumed in ref. 37 (where the current atmospheric density was used). In order to investigate NH_3_ production processes that could occur on the early Earth in less dense atmosphere, we will perform AIMD simulations with shocks with greater impact velocities as a future work.

### Formation Process of an NH_3_-N Molecule

**Figure 1:**
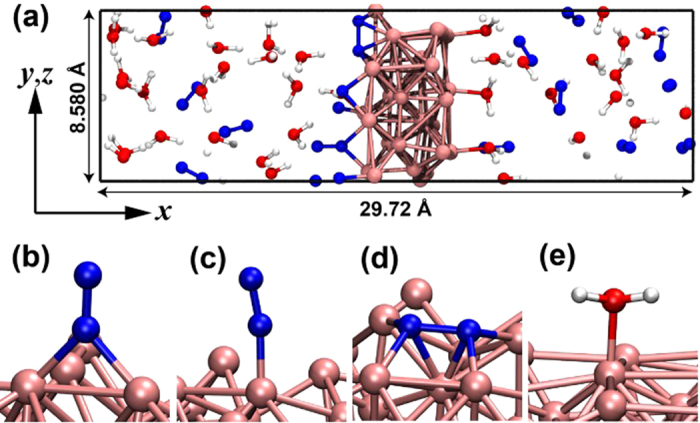
(**a**) Initial atomic configuration of the system consisting of a Fe_36_ slab, 16 N_2_, and 38 H_2_O molecules, where white, blue, red, and pink spheres represent H, N, O, and Fe atoms, respectively. (**b**–**d**) Three types of adsorption of a N_2_ molecule on the Fe slab. (**e**) Adsorption of a H_2_O molecule on the Fe slab.

Hereafter, the atomistic mechanism of the NH_3_ production observed in the 5 km/s shock-wave simulation will be described. First, the hydrogenation of one N atom by three H atoms occurs and then one ammoniacal nitrogen (NH_3_-N) molecule is formed, where the N atom not bonding to Fe atoms with three H atoms is chosen in the N_2_ molecule (see Fig. 1(b and c)). Subsequently, an NH_3_ molecule is formed by dissociation of the N-N bond. First NH_3_ molecule produced at 1.343 ps resulted from the N_2_ molecule bonded by two Fe atoms (Fig. 1(b)). Second and third ones produced at 2.044 and 3.674 ps resulted from the N_2_ molecules bonded by one Fe atom (Fig. 1(c)). In addition, the two N_2_ molecules associated with the production of NH_3_ at 1.343 and 2.044 ps were already adsorbed on the Fe slab at the beginning of simulation. The rest one was adsorbed at 3.284 ps.

**Figure 2:**
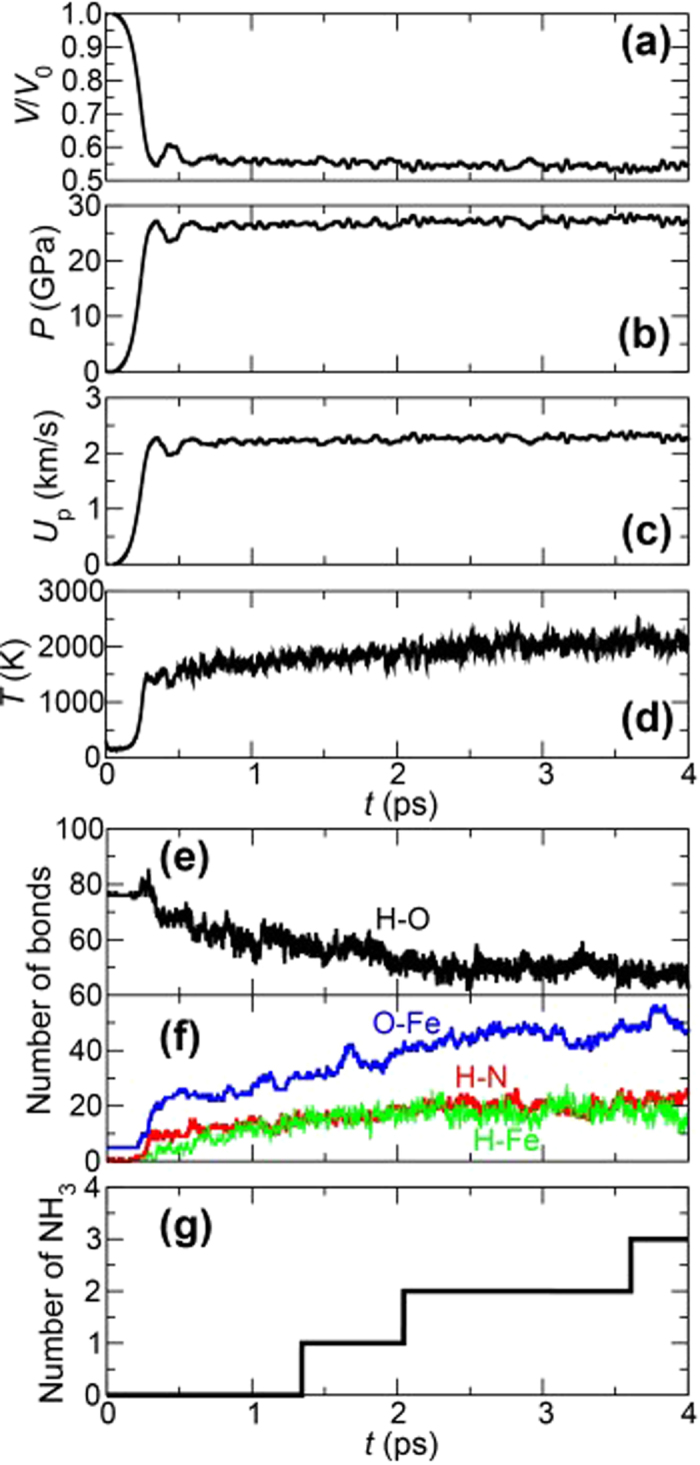
Time evolution of (**a**) volume ratio, (**b**) pressure, (**c**) particle velocity, and (**d**) temperature in 5 km/s shock-wave simulation. (**e**) and (**f**) Time evolution of the number of H-O, H-N, H-Fe, and O-Fe bonds. (**g**) Time evolution of the number of produced NH_3_.

**Figure 3:**
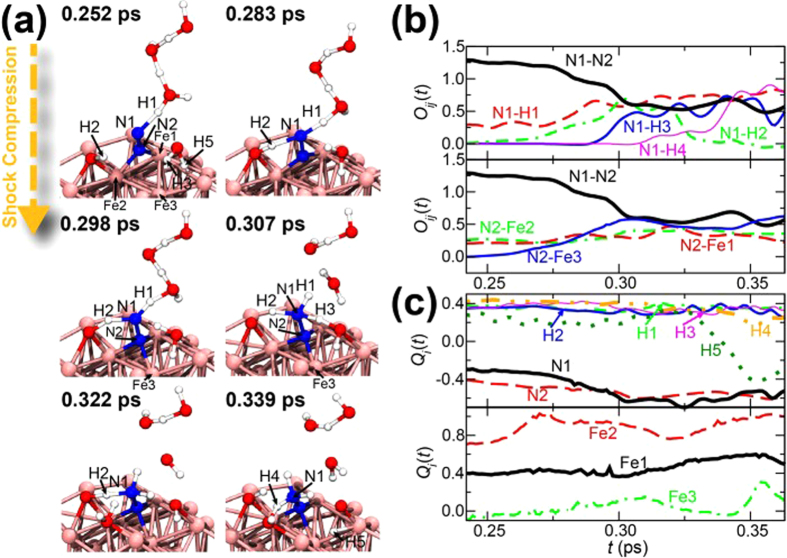
(**a**) Atomistic configurations at 0.252, 0.283, 0.298, 0.307, 0.322, and 0.339 ps during the formation of an NH_3_-N molecule on the Fe slab. Time evolution of (**b**) the bond-overlap populations *O*_*ij*_(*t*) and (**c**) the Mulliken charges *Q*_*i*_(*t*) associated with the atoms labeled in (**a**).

Figure 3 shows the formation of an NH_3_-N molecule observed in the simulation. The time evolution of the atomic configuration is shown in Fig. 3(a), where four H atoms labeled H1, H2, H3, and H4 form and break bonds with the N atom labeled N1. Figure 2(b and c) show the time evolution of the bond-overlap populations *O*_*ij*_(*t*) and the Mulliken charges *Q*_*i*_(*t*) for specified atoms using the Mulliken bond-overlap population analysis (see Methods). At 0.252 ps, a hydrogen bond of H1-N1 is formed (*O*_N1-H1_(*t*) has ~0.25) because N1 does not have neutral but negative charge (*Q*_N1_(*t*) < 0). This is because that N1 and N_2_ received some electrons from the Fe slab, leading to slight weakening of the N1-N2 bond strength (~1.3) with *Q*_N1_(*t*) and *Q*_N2_(*t*) becoming negative. Note that *O*_*ij*_(*t*) for a N-N bond of a N_2_ molecule is about 1.5 (see Supplementary Fig. S4). N2 begins to interact with Fe3 after about 0.25 ps (*O*_N2-Fe3_(*t*) increases). At 0.283 ps, H1 is transferred to N1 through the hydrogen bond, and the bonding state becomes more covalent as *O*_H1-N1_(*t*) increases to ~0.6. Subsequently, H2 of an OH fragment and H3 of a H_2_O molecule form bonds with N1 at 0.298 (*O*_N1-H2_(*t*) shows ~0.7) and 0.307 ps (*O*_N1-H3_(*t*) shows ~0.5), respectively. As *Q*_Fe2_(*t*) and *Q*_Fe3_(*t*) become more positive than those at 0.25 ps, it is considered that the supply of electrons from Fe2 and Fe3 assists to form the covalent bond of N1-H1, N1-H2, and N1-H3. In addition to these electrons, those from N1, which form a bond with N_2_, are subsequently involved in the formation of covalent bonds with H1, H2, and H3. On the other hand, the electrons from N_2_ strengthen the bond with Fe3. As a result, the N1-N2 bond weakens until 0.3 ps as *O*_N1-N2_(*t*) decreases to ~0.6. As can be seen in the snapshot at 0.307 ps, an NH_3_-N molecule consisting of N1, N2, H1, H2, and H3 is formed during the short period. It should be noted, however, that NH_3_-N molecules are not stable, thus N1 releases H2 as in the snapshot at 0.322 ps. The electronic structure of N1 becomes closed-shell when N1 forms covalent bonds with N2, H1, and H3, thus N1 probably forms a coordinate bond with H2. In this way, the bonded H atoms are easily dissociated, but other H atoms are likely to be supplied because many OH fragments and H_2_O molecules exist on the Fe slab. H4 is transferred to N1 and then an NH_3_-N molecule was formed again at 0.339 ps.

Formation of four NH_3_-N molecules was observed within 0.4 ps from adsorption of the N_2_ molecules, and three of those resulted in NH_3_. Another example of the formation of an NH_3_-N molecule is shown in Supplementary Fig. S2. Such rapid hydrogenation is due to the formation of H-rich environment driven by the following two mechanisms. One mechanism is the generation of excess H atoms arising from destabilization and strengthening of hydrogen bond networks among H_2_O molecules. This is due to shock compression that shortens the distances among the molecules^38^. In this situation, for example, adjacent two H_2_O molecules could share one H atom, and then release one H atom. The reason why H1 bonds to N1 is that the released extra H atoms induce Grotthuss-type proton hopping^39^. In addition, similar mechanism applies for the formation of H9-N3 bond at 0.295 ps in another example shown in Supplementary Fig. S2.

In the other mechanism, the adsorbed H_2_O molecules and OH fragments on the Fe slab release their H atoms such as H2 and H3. The O atoms bonding to Fe atoms release their bonding H atoms to form more bonds with the Fe atoms according to electronegativity. The sudden shock compression promotes such dissociations of H-O bonds due to pressing down the O atoms on the Fe slab surfaces. This is why the number of O-Fe bonds as shown in Fig. 2(f) rises sharply after about 0.3 ps. Note that such released H atoms often transfer to H_2_O molecules, and then the hydrogenation of N atoms occur via several H_2_O molecules by the proton hopping mechanism. For example, H10 and H11 bond to N3 and N4 via one H_2_O molecule in another example as shown in Supplementary Fig. S2.

Meanwhile, single H atom like H5 in the snapshots at 0.339 ps would hydrogenate N_2_ as well as the N atoms on the Fe slab, and we confirmed the production of a hydrazine (N_2_H_4_) molecule or hydrazinium (N_2_H_5_^+^) (the reaction process is shown in Supplementary Fig. S4). Electrons of Fe atoms are transferred to a N_2_ molecule along with proton transport among the single H atom and H_2_O molecules (see Fig. 3). This reaction is similar to the proton-coupled electron transfer (PCET) mechanism^35^,^40^. Since N_2_H_4_ is considered as an intermediate on the synthesis of NH_3_ from N_2_^35^,^41^,^42^,^43^, it would be converted to NH_3_ when higher impact velocities are given because it provides a more reducing environment.

### Dissociation Process of a N-N Bond

**Figure 4:**
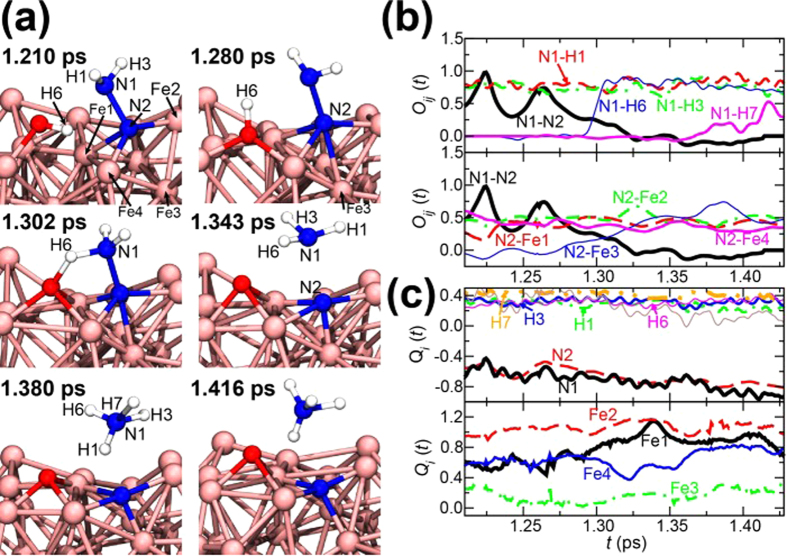
(**a**) Atomistic configurations at 1.210, 1.280, 1.302, 1.343, 1.380, and 1.416 ps during the first observed productions of an NH_3_ molecule on the Fe slab and the subsequent NH_4_^+^. Time evolution of (**b**) the bond-overlap populations *O*_*ij*_(*t*) and (**c**) the Mulliken charges *Q*_*i*_(*t*) associated with the atoms labeled in (**a**).

**Figure 5:**
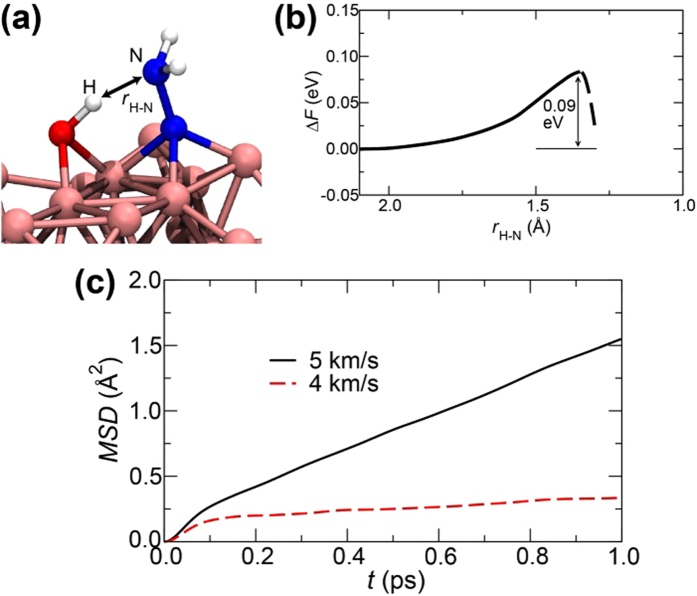
(**a**) Atomic configuration of the system of one Fe_36_ slab, one OH, and N-NH_2_ fragments picked out from the atomic configuration just before the production of the NH_3_ molecule in 5 km/s shock-wave simulation. (**b**) The free energy profile for the system as a function of the distance *r*_H-N_ between the H and N atoms labeled in (**a**). The calculation details are described in Methods. (**c**) Mean Square Displacements (*MSD*s) of Fe atoms as a function of time in 5 (solid curve) and 4 (dashed curve) km/s shock-wave simulations.

After an NH_3_-N molecule is formed, the dissociation of a N-N bond occurs. Figure 4 shows the first dissociation reaction observed in the simulation. The time evolution of the atomic configuration is shown in Fig. 4(a), where N1, N2, H1, H3, Fe1, Fe2, and Fe3 are the same ones as those in Fig. 3(a). Figure 4(b and c) show the time evolution of *O*_*ij*_(*t*) and *Q*_*i*_(*t*) for specified atoms. The snapshot at 1.210 ps represents the atomic configuration before the dissociation of N1-N2 bond, where N1 bonds to H1 and H3, and N2 bonds to Fe1, Fe2, and Fe4. After 1.210 ps, Fe3 begins to interact with N2 (*O*_N2-Fe3_(*t*) increases gradually), accompanied by electron transfer from Fe3 to the N atoms (*Q*_Fe3_(*t*) becomes positive). This leads to weakening of N1-N2 bond (*O*_N1-N2_(*t*) decreases). At around 1.3 ps, *O*_N1-H6_(*t*) increases rapidly, reflecting the fact that H6 of the adjacent OH fragment is transferred to N1. At 1.343 ps, *O*_N1-N2_(*t*) vanishes and the sum of *Q*_N1_(*t*), *Q*_H1_(*t*), *Q*_H3_(*t*), and *Q*_H6_(*t*) becomes nearly zero, *i.e.*, an NH_3_ molecule is formed. Most remarkable point is that nearly simultaneous formations of N2-Fe3 and N1-H6 bonds give rise to the dissociation of the N1-N2 bond. This requires the situation in which the N atoms bond to both H and Fe atoms. In fact, if this condition is satisfied, NH_3_ molecules could be rapidly produced. We can then evaluate the activation energy for the production of an NH_3_ molecule including the effect of finite temperatures by calculating free energies using the system consisting of the Fe_36_ slab, OH, and N-NH_2_ fragments (Fig. 5(a)) extracted from the atomic configuration just before the production of the NH_3_ molecule in 5 km/s shock-wave simulation as shown in Fig. 4(a). The calculation details are described in Methods. The estimated value of activation energy is 0.09 eV even at 300 K (see Fig. 5(b)). The corresponding reaction rates at *T* = 2,100 K (5 km/s) and 1,200 K (4 km/s) are estimated as *k* = (*k*_B_*T/h*)exp(−Δ*F/k*_*B*_*T*) = 26.6 and 10.5 ps^−1^, respectively, according to the transition state theory^44^, where *k*_B_ is the Boltzmann constant and *h* is the Planck constant. H atoms are easily transferred from the surrounding OH fragments and H_2_O molecules. Thus, we consider that the increase of mobility of Fe atoms at high temperature in 5 km/s shock-wave simulation play an essential role, *i.e.*, the temperature of 2,100 K that exceeds iron melting point of 1,810 K would provide the easier situation for N atoms to form bonds with Fe atoms. The reason why no NH_3_ was produced in 4 km/s shock-wave simulation would be that its temperature of 1,200 K is much lower than the melting point. Self-diffusion coefficients of Fe atoms *D*_Fe_ were calculated from the slopes of the mean square displacements (*MSD*s; see Fig. 4(c)) as follows:


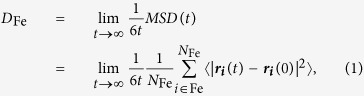


where ***r***_***i***_(*t*) and ***r***_***i***_(0) are the positions of the *i*th Fe atom at time *t* = *t* and *t* = 0, respectively, and the brackets indicate an average over Fe atoms with respect to the time origin. *N*_Fe_ is the number Fe atoms (=36). *D*_Fe_ are 2.35 × 10^−5^ and 3.01 × 10^−6^ (cm^2^/s) for 5 and 4 km/s shock-wave simulations, respectively. The former is about one order magnitude larger than the latter, and corresponds to that in molten iron. In other two production processes of NH_3_, the formation of a N-Fe bond before dissociation of a N-N bond is a common feature (one example is shown in Supplementary Fig. S4).

The produced NH_3_ at 1.343 ps immediately receives one H atom from a neighboring H_3_O^+^ (*O*_N1-H7_(*t*) > 0), and then becomes an NH_4_^+^. All the produced NH_3_ prefer to exist as NH_4_^+^ under the condition of the present simulation.

Although Nakazawa *et al*.^24^ assumed the Haber-Bosch process, the NH_3_ production processes observed in the present simulation correspond to the reduction of N_2_ via the associative mechanism as seen in the synthesis catalyzed by nitrogenase enzyme^34^,^35^. Reduction of N_2_ by the Haber-Bosch reaction follows the dissociative mechanism, where the N-N bond is dissociated before hydrogenation^34^,^35^. However, since the single H and N atoms exist on the Fe slab, the NH_3_ production via the Haber-Bosch process can happen only if the long-term simulation is performed.

## Discussion

In summary, our MSST-AIMD simulations revealed rapid NH_3_ production in shocked simple system consisting of metallic iron, N_2_, and H_2_O, imitating the prebiotic Earth during the LHB periods. One key factor is the rapid hydrogenation of N atoms on the Fe slab. Due to shock compression, excess H atoms are released from densified H_2_O molecules and those adsorbed on the Fe slab. The released H atoms are likely to be transferred to the N atoms directly or by Grotthuss-type proton hopping mechanism. Assisted by electron transfer from Fe to N atoms, the associated H atoms form covalent bonds with the N atoms. For the subsequent N-N bond dissociation, increase in mobility of the Fe atoms due to high temperature beyond its melting point would facilitate the formation of N-Fe bonds. The observed NH_3_ production processes have characteristics in common with the associative mechanism as seen in the catalysis of nitrogenase enzyme. We also found that a N_2_H_4_ molecule was produced through the reduction of a N_2_ molecule by transferring the dissociated electron-rich H atoms on the Fe slab via H_2_O molecules. It is therefore concluded that shocks with greater impact velocities would achieve the rapid NH_3_ production and increase in the yield of its product.

Considering also CO_2_ which is one of the main components of the prebiotic atmosphere^3^,^4^, we believe that not only NH_3_ but also some reduced carbon sources were formed in the early stage during shock compression. Even for the ironless system consisting of CO_2_ and H_2_O, the precursors of a formic acid were obtained during shock compression in the classical MD simulation^38^. Also, a recent AIMD study revealed that considerably larger amounts of C-C and C-H bonds were formed at high pressure and temperature in the system consisting of Fe atoms, CO_2_, and H_2_O than the system without Fe atoms^45^. In the MD studies by Goldman *et al*.^32^,^33^ which demonstrated the formation of glycine and important precursors of biomolecules, NH_3_ and CH_3_OH were used as starting materials because they assumed cometary components. However, if such reduced nitrogen and carbon sources can be produced from terrestrial molecules in the early stage of Earth during shock compression, the meteorites including metallic iron would also provide a similar result obtained in the case of the comet. The possibility would be high, taking into account that the recent shock experiments by Nakazawa *et al*.^25^ demonstrated the production of a variety of amino acids and nucleobases in shocked sample including metallic iron. Anyway, since we have made an investigation only for limited conditions, further intensive studies should be needed.

Lastly, we note that the shock-induced NH_3_ production might have also occurred on ancient Mars. The previous studies have suggested that the early Martian atmosphere contained N_2_^46^ and a vast ancient ocean existed^47^ during the LHB periods. Although the yield is considered to be smaller compared to that on Earth because of the rarefied Martian atmosphere, we suppose that the NH_3_ production mechanism reported in this study could be a probable model for providing NH_3_ on Mars as well as on Earth.

## Methods

We simulated the system consisted of a Fe_36_ slab, 16 N_2_, and 38 H_2_O molecules (a total of 182 atoms; see Fig. 1(a)). A rectangular supercell of dimensions 29.72 Å × 8.580 Å × 8.580 Å under periodic boundary conditions was employed. Quasi-Newton method^48^ was used for structural optimization to prepare an initial atomic configuration. Using this atomic configuration, we performed two multi-scale shock technique-based *ab initio* molecular dynamics (MSST-AIMD) simulations, in which shock waves propagated in the *x* direction with shock speeds of 5 and 4 km/s. In our MSST-AIMD simulations, electronic states were calculated using the projector-augmented-wave (PAW) method^31^,^49^. Projector functions were generated for the 2s and 2p states of N and O atoms, the 1 s state for H, and the 3d, 4s, and 4p states of Fe atoms. The generalized gradient approximation^50^ was used for the exchange-correlation energy with non-linear core corrections^51^, along with van der Waals correction based on the DFT-D method^52^. The spin polarization effects were neglected. The momentum-space formalism^53^ was utilized, where the plane-wave cutoff energies were 30 and 250 Ry for the electronic pseudo-wave functions and the pseudo-charge density, respectively, and the Γ point was used in the Brillouin zone. The energy functional was minimized iteratively using a preconditioned conjugate-gradient method^54^,^55^. MSST^30^ was used to simulate a steady shock wave by augmenting the equations of motion of atoms with dynamically evolving the volume of the computational cell, while constraining the stress to the Rayleigh line and the energy to the Hugoniot relation^56^. The dynamics of the system is governed by the extended Lagrangian,


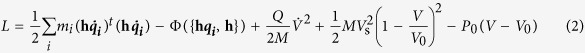


where *m*_*i*_ is the mass of the *i*th atom, ***q***_***i***_ is a column vector whose components are the *i*th atom’s scaled coordinates in the range of [0, 1], Φ is the potential energy, *Q* is a parameter with unit of (mass)^2^ · (length)^−4^, *M* = ∑_*i*_ *m*_*i*_ is the total mass of the system, and *V*_s_ is the speed of the shock wave. The real coordinate and the velocity of the *i*th atom are given by **h*****q***_***i***_ and 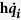 respectively, where **h** = (***L***_1_ ***L***_2_ ***L***_3_) is a matrix consisting of the computational cell lattice vectors ***L***_***k***_ (*k* = 1, 2, 3). *V* = det **h** is the volume of the computational cell. *P*_0_ and *V*_0_ = det **h**_0_ are the pressure and volume of the unshocked state, respectively, where **h**_0_ corresponds to **h** in the unshocked state. In equation (2), a dot denotes time derivative. Initial pressure and temperature were set to 0 GPa and 300 K, respectively. The equations of motion were integrated numerically with a time step of 10 a.u. (=0.242 fs). Simulations were performed for the time duration of 4 ps.

We used population analysis^57^,^58^ to clarify the changes in the bonding properties of atoms associated with the production processes of NH_3_. By expanding the electronic wave functions in an atomic-orbital basis set^59^,^60^, we obtained the bond-overlap population (BOP or *O*_*ij*_(*t*)) between *i*th and *j*th atoms and the gross population *Z*_*i*_(*t*) for *i*th atom, which are based on a formulation generalized to the PAW method^61^. The Mulliken charge *Q*_*i*_(*t*) was then obtained as the difference between the number of valence electrons of an isolated neutral atom 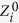
(*t*) and the value of the gross population *Z*_*i*_(*t*):


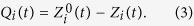


*O*_*ij*_(*t*) gives a semi-quantitative estimate of the strength of covalent bonds between atoms, and we estimated the charges of the atoms from Q_*i*_(*t*). The charge spillage, which estimates the error in the expansion, was only about 0.6 %, indicating the high quality of the atomic-orbital basis.

We evaluated the activation energy for the production of an NH_3_ molecule including the effect of finite temperatures by calculating free energies. For this purpose, additional AIMD simulations were performed at *T* = 300 K by imposing geometrical constraints to obtain the free energy profile^62^ along the NH_3_ production reaction path. The Lagrange multiplier 〈*λ*〉 was introduced to constrain the distance *r*_H-N_ between one H and one N atoms to be reacted. By taking time average, we obtained the average Lagrange multiplier 〈*λ*〉. The canonical ensemble simulation using the Nosé-Hoover thermostat technique^63^ was performed for 1 ps at each distance *r*_H-N_. The 〈*λ*〉 becomes zero at an equilibrium distance *r*_0_. The value of *r*_H-N_ is decreased from this distance, and again 〈*λ*〉 becomes zero at the distance *r*_d_ at which an NH_3_ molecule is produced. The relative free energies Δ*F* were obtained for *r*_0_ > *r*_H-N_ > *r*_d_ by the following integral^64^:


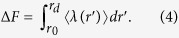


We calculated the free energy profiles along the corresponding reaction path using the system consisting of a Fe_36_ slab, an OH, and a N-NH_2_ fragments (see Fig. 5(a)) picked out from the atomic configuration just before the production of the NH_3_ molecule in 5 km/s shock-wave simulation as shown in Fig. 4(a).

## Additional Information

**How to cite this article:** Shimamura, K. *et al*. Meteorite Impact-Induced Rapid NH_3_ Production on Early Earth: *Ab Initio* Molecular Dynamics Simulation. *Sci. Rep.* **6**, 38953; doi: 10.1038/srep38953 (2016).

**Publisher's note:** Springer Nature remains neutral with regard to jurisdictional claims in published maps and institutional affiliations.

## Supplementary Material

Supplementary Information
