# Supplementary material for: Meteorite Impact-Induced Rapid NH3 Production on Early Earth: Ab Initio Molecular Dynamics Simulation
Source: Sci Rep. 2016 Dec 14;6:38953. doi: 10.1038/srep38953 (PMC5155216; doi:10.1038/srep38953)
Supplement: Supplementary Information [file srep38953-s1.pdf]

Supplementary Information for "Meteorite Impact-Induced Rapid NH_3 Production on Early Earth: *Ab Initio* Molecular Dynamics Simulation"

Kohei Shimamura[1], Fuyuki Shimojo[2], Aiichiro Nakano[3], and Shigenori Tanaka[1]

[1]*Graduate School of System Informatics, Kobe University, Kobe, 657-8501, Japan*

[2]*Department of Physics, Kumamoto University, Kumamoto 860-8555, Japan*

[3]*Collaboratory for Advanced Computing and Simulations, Department of Physics & Astronomy, Department of Computer Science, Department of Chemical Engineering & Materials Science, University of Southern California, Los Angeles, CA 90089-0242, USA*

<u>Rough Estimate of Production Amount of NH_3 Using the Results of Shock Experments by Nakazawa *et al*.</u>

The shock experiments by Nakazawa *et al.*[1] have confirmed the production of NH_3 and the nitrogen conversion rate of 8 % was obtained. Based on the conversion rate, the amount of NH_3 produced during the heavy bombardment can be estimated as about 1.08×10^{7} tons annually. Here, the estimated amount of meteorite accretion 4×10^{24} g during 4.4 to 3.8 billion years ago[2] is used. It is also assumed that the whole accretion occurred by ordinary chondrites contained 10 wt.% iron[3] (more than 85 % of recovered meteorites are classified as ordinary chondrites[4]), and the iron is totally consumed for the NH_3 production through the reaction: $N_2 + 3H_2O + 3Fe \rightarrow 2NH_3 + 3FeO$.

Time Evolution of Physical Quantities and Number of Bonds in 4 km/s Shock-Wave Simulation

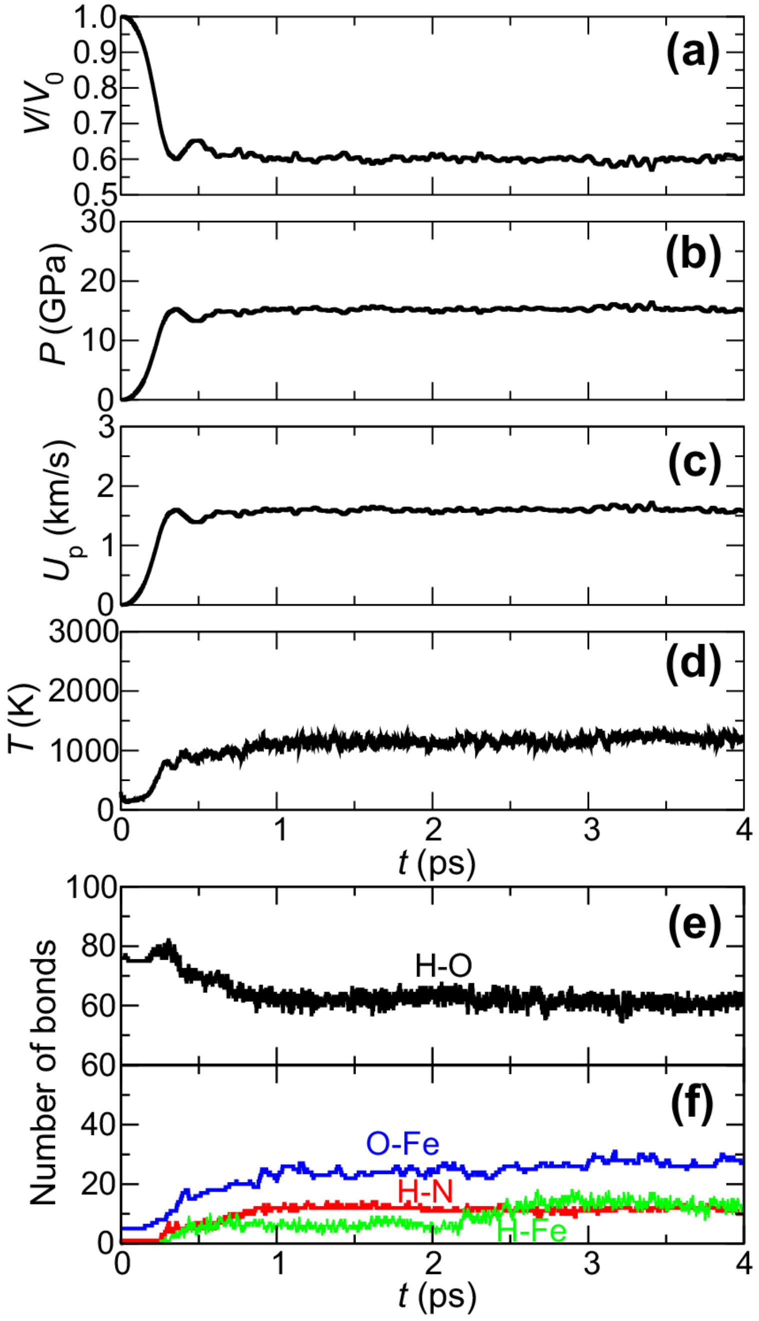

Figure S1. Time evolution of (a) volume ratio, (b) pressure, (c) particle velocity, and (d) temperature in 4 km/s shock-wave simulation. (e) and (f) Time evolution of the number of H-O, H-N, H-Fe, and O-Fe bonds.

Rough Estimate of Production Amount of NH_3 Using the MSST-AIMD Simulation Results

From previous simulation studies[5-7], time scale of shock compression is assumed to be 10 ps. In our simulation (5 km/s shock-wave simulation), three NH_3 molecules were produced on the surface of Fe_{36} slab during 4 ps, *i.e.*, about 0.21 NH_3 molecules are produced per Fe atom during 10 ps. The estimated amount of meteorite accretion 4×10^{24} g during 4.4 to 3.8 billion years ago[2] is also used. Assuming that the whole accretion occurred by ordinary chondrites contained 10 wt.% iron[3], Fe atoms of about 1.2×10^{13} mol were annually supplied to the early Earth during 4.4 to 3.8 billion years ago. We therefore consider that NH_3 of about 2.5×10^{12} mol yr^{-1} (or 4.3×10^{7} tons yr^{-1}) were produced. It should be noted that the amount is larger than 1.08×10^{7} tons yr^{-1} estimated from the experimental result by Nakazawa *et al.*[1], which corresponds to our lower shock-energy simulation (4 km/s shock-wave simulation). This implies that shocks with greater impact velocities would provide further increase in the yield if all of them can survive after quenching.

If we assume that the volume of early sea is 10^{21} L [8] and all of the NH_3 produced during shock compression (about 2.5×10^{12} mol yr^{-1}) are dissolved into the sea, the concentration of NH_3 in the early sea can be estimated as about 2.5×10^{-8} mol/L, which is much smaller than that in other proposed mechanisms such as reductions of NO^{2-} and NO^{3-} by oceanic Fe^{2+} and of crustal N_2 on the mineral surfaces around submarine hydrothermal systems (7×10^{-5} - 8×10^{-7} mol/L)[9]. However, we emphasize that meteorite impacts would form locally NH_3-enriched areas in the early ocean, and the produced NH_3

would be directly involved in the subsequent amino acid production reaction, taking account of the MSST-AIMD results by Goldman *et al.*[10] Thus, the discussion above of the average concentration on the early ocean may not be relevant in the Earth-science context.

Second Production Process of an NH_3 molecule

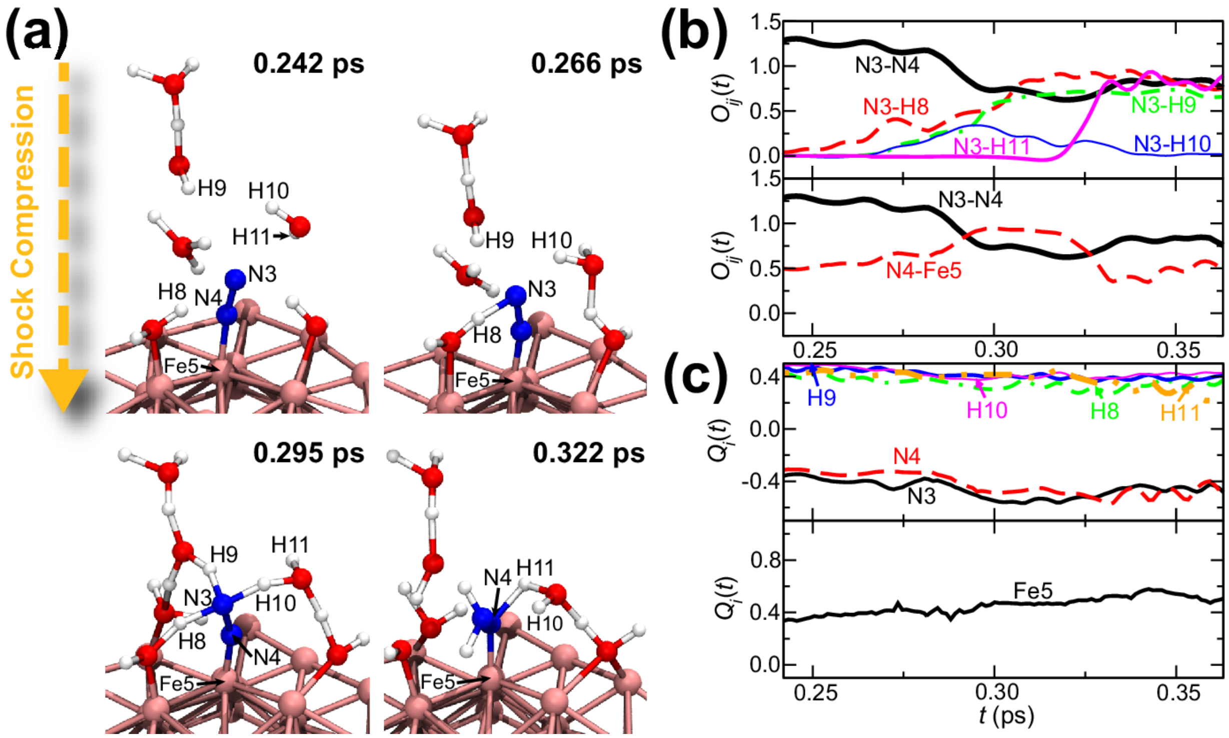

Figure S2. (a) Atomistic configurations at 0.242, 0.266, 0.295, and 0.322 ps during the formation of an ammoniacal nitrogen (NH_3-N) molecule on the Fe slab. Time evolution of (b) the bond-overlap populations $O_{ij}(t)$ and (c) the Mulliken charges $Q_i(t)$ associated with the atoms labeled in (a).

Figure S2 shows another example of the formation of an ammoniacal nitrogen (NH_3-N) molecule observed in 5 km/s shock-wave simulation. The time evolution of the atomic configuration is shown in Figure S2(a), where four H atoms labeled H8, H9, H10, and H11 form and break bonds with the N atoms labeled N3 and N4. Figures S2(b) and S2(c) show the time evolution of the bond-overlap populations $O_{ij}(t)$ and the Mulliken charges $Q_i(t)$ for specified atoms using the Mulliken bond-overlap population analysis.

At 0.242 ps, N4 bonds to Fe5 ($O_{N4\text{-}Fe5}(t)$ shows ~0.5), and this leads to weakening the strength of N3-N4 bond ($O_{N3\text{-}N4}(t)$ shows ~1.25), and $Q_{N3}(t)$ and $Q_{N4}(t)$ have negative

charges. At the same time, N3 begins to interact with H8 as $O_{N3\text{-}H8}(t)$ increases gradually. By the increase in density of H_2O molecules due to shock compression, N3 also begins to interact with H9 and H10 at 0.266 ps. An NH_3-N molecule consisting of N3, H8, H9, and H10 is formed at 0.295 ps. While N3 forms bonds with the H atoms, N4-Fe5 bond is strengthened as $O_{N4\text{-}Fe5}(t)$ reaches ~0.9, accompanied by the increase in positive charge for $Q_{Fe5}(t)$. In contrast, since $O_{N3\text{-}N4}(t)$ decreases to ~0.75, the N3-N4 bond strength is weakened.

However, H10 does not form a stable bond with N4. Thus, $O_{N4\text{-}H10}(t)$ begins to decrease after 0.295 step, while $O_{N3\text{-}H8}(t)$ and $O_{N3\text{-}H9}(t)$ continue to increase. Instead, N4 begins to interact with H11 at around 0.315 ps. $O_{N4\text{-}H11}(t)$ increases to ~0.8 at 0.322 ps. On the other hand, $O_{N4\text{-}Fe5}(t)$ rapidly decreases to ~0.5. Also, $O_{N3\text{-}N4}(t)$ slightly increases to ~0.85.

Figure S3 shows the second dissociation reaction of N-N bond observed in 5 km/s shock-wave simulation. The time evolution of the atomic configuration is shown in Figure S3(a), where N3, N4, H9, H10, and Fe5 are the same atoms as those in Figure S2(a). Figures S3(b) and S3(c) show the time evolution of $O_{ij}(t)$ and $Q_i(t)$ for specified atoms. Up to 2.021 ps, three and two H atoms have bonded to N3 and N4, respectively. Although $O_{N3\text{-}N4}(t)$ shows ~0.55 at this time, it begins to decrease gradually because N3 also begins to interact with Fe6. At 2.059 ps, $O_{N3\text{-}N4}(t)$ intersects with $O_{N4\text{-}Fe6}(t)$, and then it reaches zero and the sum of $Q_{N3}(t)$, $Q_{H9}(t)$, $Q_{H10}(t)$, and $Q_{H12}(t)$ becomes nearly equal to zero, *i.e.*, an NH_3 molecule is produced.

Subsequently, H15 is supplied to N3 from an OH fragment on the Fe slab at around 2.142 ps, which indicates that an NH_4^+ is formed.

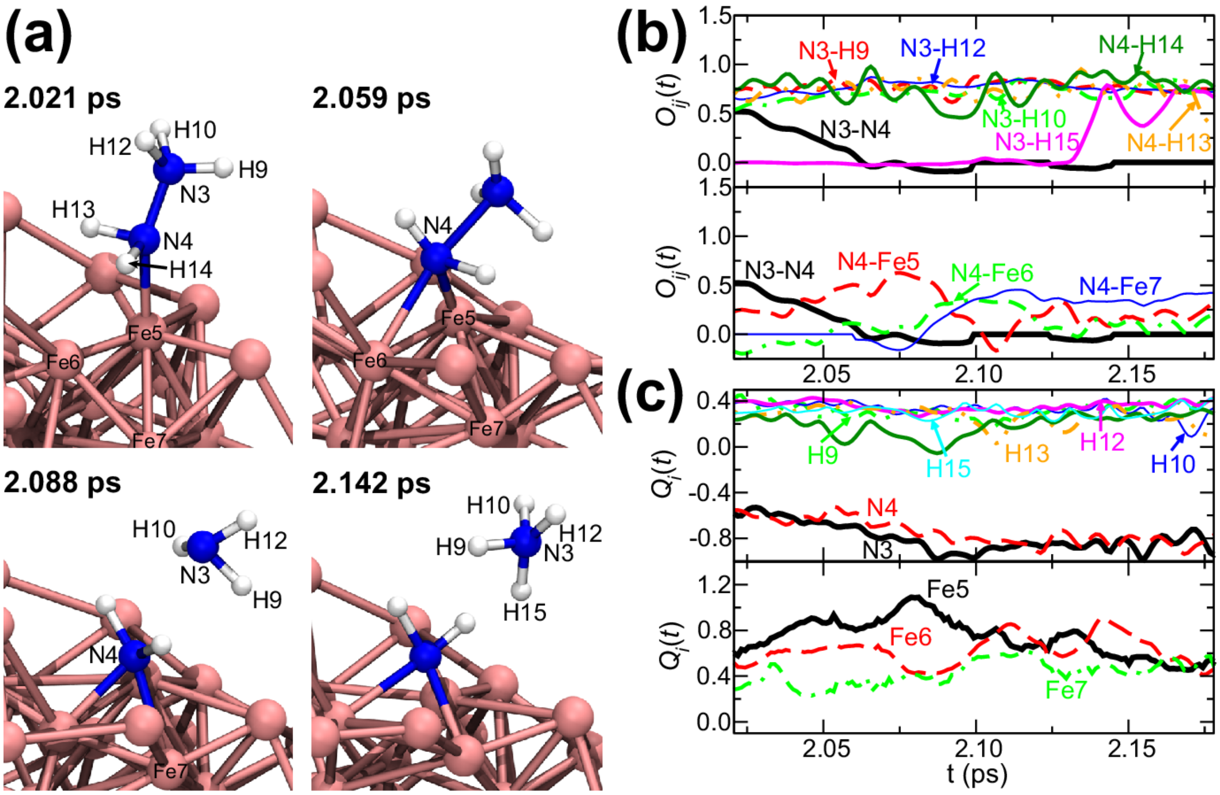

Figure S3. (a) Atomistic configurations at 2.021, 2.059, 2.088, and 2.142 ps during the second observed production of an NH_3 molecule on the Fe slab and the subsequent NH_4^{+}. Time evolution of (b) the bond-overlap populations $O_{ij}(t)$ and (c) the Mulliken charges $Q_i(t)$ associated with the atoms labeled in (a).

Production Process of a Hydrazinium ($N_2H_5^+$)

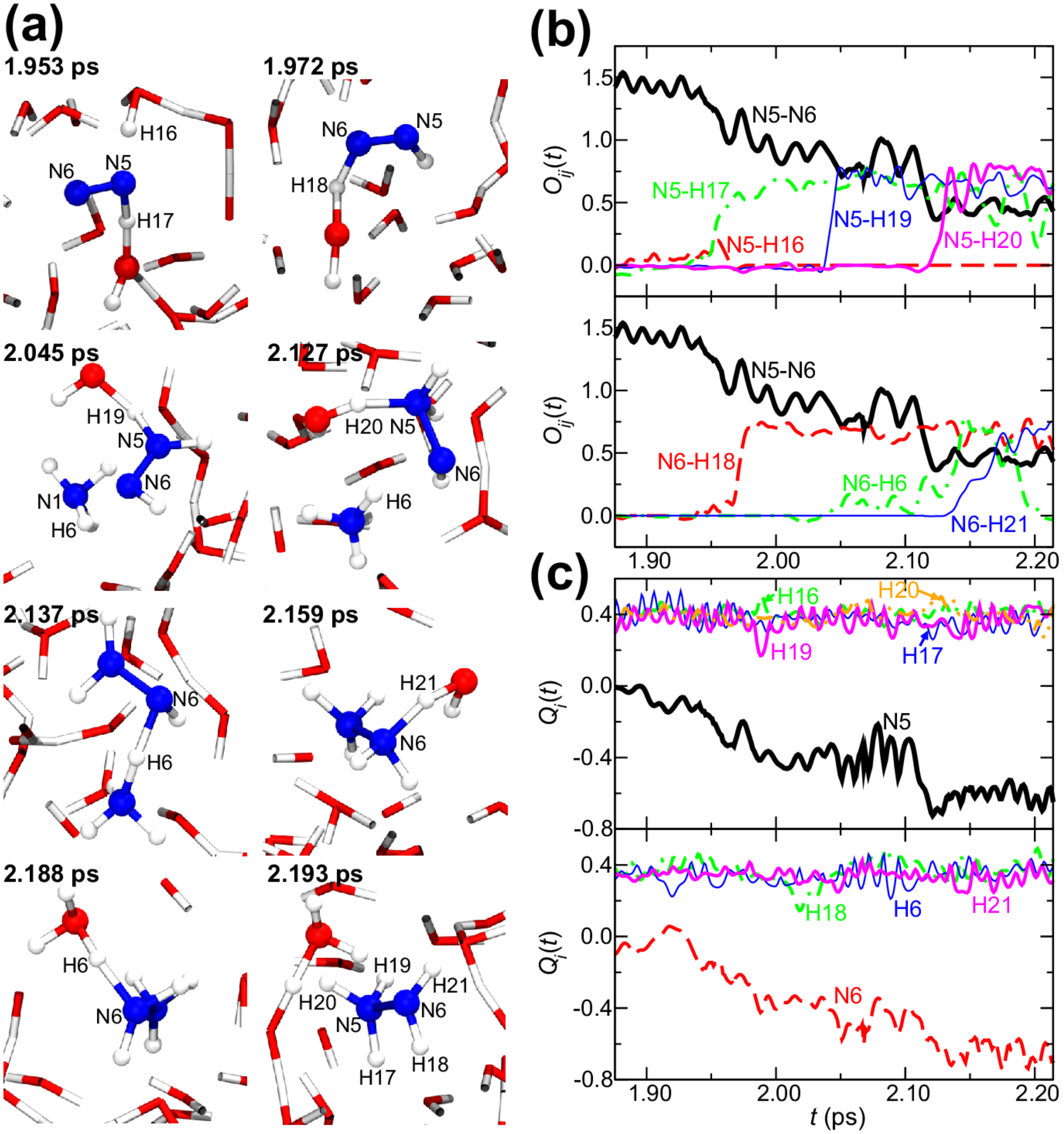

Figure S4. (a) Atomistic configurations at 1.953, 1.972, 2.045, 2.127, 2.137, 2.159, 2.188, and 2.193 ps during the production of a hydrazinium ($N_2H_5^+$) from a N_2 molecule. Time evolution of (b) the bond-overlap populations $O_{ij}(t)$ and (c) the Mulliken charges $Q_i(t)$ associated with the atoms labeled in (a).

Figure S4 shows the production reaction of a hydrazinium ($N_2H_5^+$) observed in 5 km/s shock-wave simulation. The time evolution of the atomic configuration is shown in Figure S4(a). Figures S4(b) and S4(c) show the time evolution of $O_{ij}(t)$ and $Q_i(t)$ for specified atoms. At 1.876 ps, a N_2 molecule consisting of N5 and N6 exists in water. $Q_{N5}(t)$ and $Q_{N6}(t)$ have nearly neutral charges, and $O_{N5\text{-}N6}(t)$ has ~1.5 which indicates the strength of a triple bond. As $O_{N5\text{-}H16}(t)$ has a positive finite value, N5 has formed a hydrogen bond with H16 of the neighbor H_2O molecule up to 1.88 ps. This bond formation makes $Q_{N5}(t)$ slightly more negative, and leads to the hydrogen bond formation between N5-H17 at about 1.94 ps. Subsequently, H17 forms a covalent bond with N5 as $O_{N5\text{-}H17}(t)$ rapidly increases after 1.953 ps.

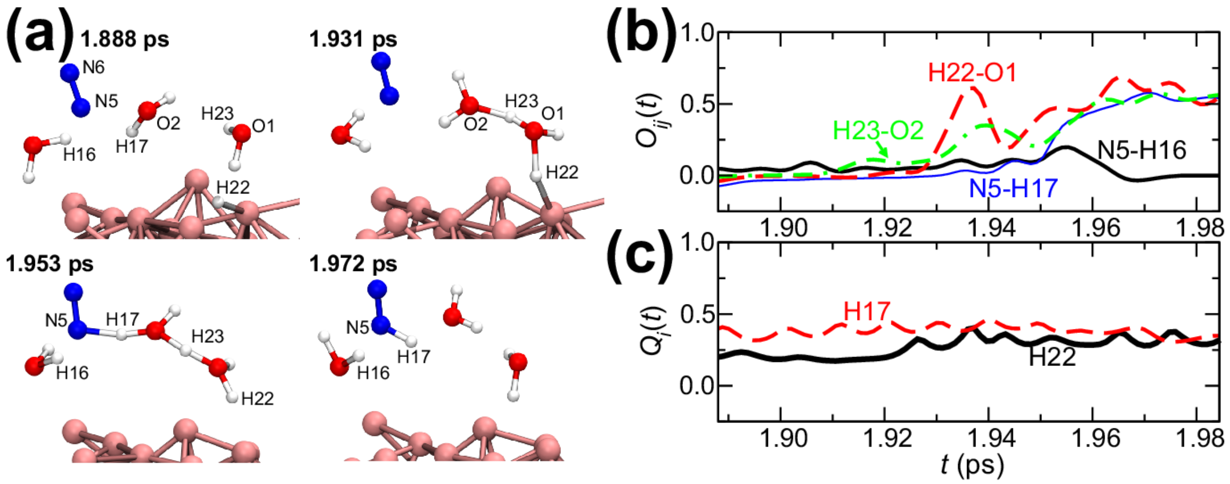

Figure S5. (a) Atomistic configurations at 1.888, 1.931, 1.953, and 1.972 ps during the bonding process of H17 to N5 via electron transfer from the Fe slab. Time evolution of (b) the bond-overlap populations $O_{ij}(t)$ and (c) the Mulliken charges $Q_i(t)$ associated with the atoms labeled in (a).

It should be noted that the used electron to form the covalent bond is supplied from Fe atoms. Figure S5 shows the formation process of a covalent-bond H17-N5. H22 is one of the dissociated H atoms from a H_2O molecule existing on the Fe slab surface (see the

number of H-Fe bonds shown in Figure 2(f) of the main text). In contrast to the charges of H atoms in H_2O molecules (*e.g.* $Q_{H17}(t)$), $Q_{H22}(t)$ has much more negative charge at 1.888 ps because it receives an electron from Fe atoms. In addition, two H_2O molecules exist between H22 and the N_2 molecule. Along with the transport of H17 to O1 at 1.931 ps, the interatomic distances between H22-O1, H23-O2, and N5-H17 are shortened and the hydrogen bonds are formed. As a result, since the electron of H22 as well as H17 itself are transferred to N5 via the hydrogen bond network, the covalent bond between N5 and H17 is formed at 1.953 ps. It is considered that this reaction is similar to the proton-coupled electron transfer (PCET) mechanism[11,12].

Such electron and H-atom transfers via the hydrogen bond networks provide the consecutive hydrogenation of the N_2 molecule. At around 1.972 ps, H18 has a covalent interaction with N6, and this gives rise to weakening N5-N6 bond. $O_{N5\text{-}N6}(t)$ has ~1.0 which indicates that the bond becomes a double bond, and the sum of $Q_{N5}(t)$, $Q_{N6}(t)$, $Q_{H17}(t)$, and $Q_{H18}(t)$ becomes nearly zero, *i.e.*, a diazene (N_2H_2) molecule is produced. Although the formations of the N5-H19 and N6-N6 bonds occur at about 2.057 ps, these are not covalent but the former is a coordinate bond using a lone pair of N5 and the latter is a hydrogen bond, considering that $O_{N5\text{-}N6}(t)$ maintains ~1.0 and $Q_{N5}(t)$ and $Q_{N6}(t)$ does not decrease from ~-0.4. Note that H6 belongs to a neighboring NH_4^+, which is the same H atom labeled H6 as shown in Figure 4(a) of the main text. After about 2.127 ps, N5 begins to interact with H20. Simultaneously N6-H6 bond is slightly enhanced, while N5-N6 bond begins to weaken. At about 2.137 ps, $Q_{N5}(t)$ and $Q_{N6}(t)$ reach ~-0.6, and $O_{N5\text{-}N6}(t)$ decreases to ~0.5 which indicates that the bond becomes a single covalent bond. Thus, a hydrazinium ($N_2H_5^+$) is formed although $O_{N6\text{-}H6}(t)$ shows a smaller value (~0.25) at this

time than those of $O_{N5-H17}(t)$, $O_{N5-H19}(t)$, $O_{N5-H20}(t)$, and $O_{N6-H18}(t)$ which maintain ~0.7. Furthermore, while the strength of N6-H6 bond is enhanced to ~0.75 afterwards, N6 forms a bond with H21 as $O_{N6-H21}(t)$ has a positive finite value at 2.159 ps. This indicates that a hydrazinium(2+) ($N_2H_6^{2+}$) is formed. However, this is an instantaneous formation, and then H6 begins to interact with a neighboring H_2O molecule at 2.188 ps. Subsequently, $O_{N6-H6}(t)$ disappears at around 2.20 ps, accompanying the increase in $O_{N6-H21}(t)$ to ~0.7. Since $O_{ij}(t)$ for the five N-H bonds (N5-H17, N5-H19, N5-H20, N6-H18, and N6-H21) show large values ~0.7 as well as the sum of $Q_{N5}(t)$, $Q_{N6}(t)$, $Q_{H17}(t)$, $Q_{H18}(t)$, $Q_{H19}(t)$, $Q_{H20}(t)$, and $Q_{H21}(t)$ becomes nearly +1, a stable $N_2H_5^+$ is formed.

References for Supplementary Information

1. Nakazawa, H., Sekine, T., Kakegawa, T. & Nakazawa, S. High yield shock synthesis of ammonia from iron, water and nitrogen available on the early Earth. *Earth Planet. Sci. Lett.* **235**, 356–360 (2005).

2. Kasting, J. Bolide Impacts and the Oxidation-State of Carbon in the Earth's Early Atmosphere. *Orig. Life Evol. Biosph.* **20**, 199–231 (1990).

3. Afiattalab, F. & Wasson, J. T. Composition of the metal phases in ordinary chondrites: implications regarding classification and metamorphism. *Geochim. Cosmochim. Ac.* **44**, 431 – 446 (1980).

4. Norton, O. *The Cambridge Encyclopedia of Meteorites* (ed. Norton, O.) 331–340 (Chambridge Univ. Press, 2002).

5. Robertson, D. H., Brenner, D. W. & White, C. T. Split shock wave from molecular dynamics. *Phys. Rev. Lett.* **67**, 3132-3135 (1991).

6. Gahagan, K. T., Moore, D. S., Funk, D. J., Rabie, R. L. & Buelow, S. J. Measurement of shock wave rise times in metal thin films. *Phys. Rev. Lett.* **85**, 3205-3208 (2000).

7. Kadau, K., Germann, T. C., Lomdhal, P. S. & Holian, B. L. Microscopic view of structural phase transitions induced by shock waves. *Science* **296**,1681-1684 (2002).

8. Aubrey, A. D., Cleaves, H. J., & Bada, J. L. The role of submarine hydrothermal systems in the synthesis of amino acids. *Orig. Life Evol. Biosph.* **39**, 91-108 (2009).

9. Furukawa, Y., Nakazawa, H., Sekine, T., Kobayashi, T. & Kakegawa, T. Nucleobase and amino acid formation through impacts of meteorites on the early ocean. *Earth Planet. Sci. Lett*. **429**, 216‑222 (2015).

10. Goldman, N., Reed, E. J., Fried, L. E., Kuo, I. F. W. & Maiti, A. Synthesis of glycine-containing complexes in impacts of comets on early Earth. *Nat. Chem*. **2**, 949‑954 (2010).

11. Saveant, J.-M. Electrochemical approach to proton-coupled electron transfers: recent advances. *Energy Environ. Sci.* **5**, 7718–7731 (2012).

12. van der Ham, C. J. M., Koper, M. T. M. & Hetterscheid, D. G. H. Challenges in reduction of dinitrogen by proton and electron transfer. *Chem. Soc. Rev*. **43**, 5183–5191 (2014).
